# Associação entre o Uso de Fondaparinux com Acesso Radial e Desfechos Clínicos em Pacientes com Síndrome Coronariana Aguda sem Supradesnivelamento do Segmento ST

**DOI:** 10.36660/abc.20240329

**Published:** 2025-11-13

**Authors:** Luiz Eduardo Fonteles Ritt, Eduardo Sahade Darze, Pedro Gabriel Melo de Barros e Silva, Gilson Soares Feitosa-Filho, João Victor Santos Pereira Ramos, Márcia A. Viana, Priscila Neri Lacerda, Emanoela Lima Freitas, Queila Oliveira Borges, Adriano Oliveira Martins, Renato Delascio Lopes

**Affiliations:** Instituto D’Or de Pesquisa e Ensino Hospital Cardio Pulmonar BA Brasil Instituto D’Or de Pesquisa e Ensino, Hospital Cardio Pulmonar, BA – Brasil; 2 Escola Bahiana de Medicina e Saúde Pública Salvador BA Brasil Escola Bahiana de Medicina e Saúde Pública, Salvador, BA – Brasil; 3 Hospital Samaritano Paulista São Paulo SP Brasil Hospital Samaritano Paulista, São Paulo, SP – Brasil; 4 Universidade Federal da Bahia Salvador BA Brasil Universidade Federal da Bahia, Salvador, BA – Brasil; 5 Duke University Hospital Durham North Carolina EUA Duke University Hospital, Durham, North Carolina – EUA

**Keywords:** Síndrome Coronariana Aguda, Cateterismo Cardíaco, Prognóstico

## Abstract

**Fundamento:**

Tanto o fondaparinux quanto o acesso radial têm sido associados a menores taxas de eventos adversos cardiovasculares maiores (MACE) em pacientes com síndrome coronariana aguda (SCA).

**Objetivo:**

Avaliar a associação entre o uso de fondaparinux combinado ao acesso radial e os desfechos clínicos.

**Métodos:**

Foram analisados 956 pacientes internados com SCA e submetidos a estratégia invasiva. O desfecho primário — composto por sangramento maior (segundo os critérios do estudo OASIS-5) e MACE — foi comparado entre os grupos definidos conforme o esquema anticoagulante (fondaparinux ou enoxaparina) e o sítio de acesso arterial (femoral ou radial). Valores de p < 0,05 foram considerados estatisticamente significativos.

**Resultados:**

A média de idade da população foi de 65 ± 12,4 anos, sendo que 49,5% apresentavam infarto agudo do miocárdio sem supradesnivelamento do segmento ST (IAMSSST). O uso de fondaparinux + acesso radial foi observado em 366 pacientes. O desfecho primário ocorreu em 78 pacientes (8,1%), sendo MACE em 50 (5,2%) e sangramento maior em 32 (3,3%). A menor taxa de eventos foi observada no grupo fondaparinux + acesso radial (3,3%), em comparação com enoxaparina + acesso radial (9,8%), fondaparinux + femoral (8,6%) e enoxaparina + femoral (14,4%) (p < 0,001). Na análise multivariada, o uso de fondaparinux foi associado a uma redução de 43% no desfecho primário (OR 0,57; IC 95% 0,34-0,96; p < 0,05), e o acesso radial foi independentemente associado a uma redução de 54% (OR 0,46; IC 95% 0,26-0,83; p = 0,01).

**Conclusão:**

A combinação de fondaparinux com acesso radial foi associada às menores taxas de MACE e sangramento maior, quando comparada a cada uma das estratégias isoladamente.

**Figure:**
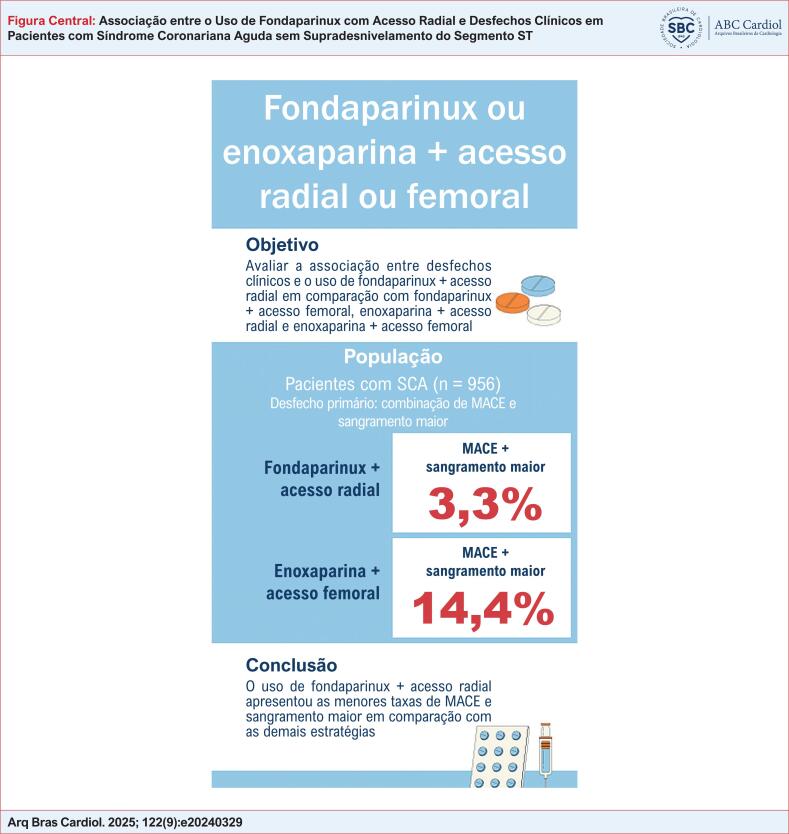
MACE: eventos adversos cardiovasculares maiores; SCA: sídrome coronariana aguda.

## Introdução

As síndromes coronarianas agudas (SCA) estão entre as principais causas de mortalidade em todo o mundo. Nas últimas duas décadas, o tratamento das SCA evoluiu para incorporar uma abordagem agressiva com a combinação de anticoagulantes e agentes antiplaquetários, associada a uma estratégia invasiva precoce, com a realização de cinecoronariografia e intervenção coronariana percutânea (ICP), quando indicada. Como resultado, o risco de complicações isquêmicas foi significativamente reduzido; no entanto, observou-se um aumento proporcional no risco de sangramentos.^1–3^

Eventos hemorrágicos em pacientes com SCA estão associados a piores desfechos clínicos, incluindo aumento da mortalidade. Por esse motivo, o risco de sangramento tornou-se um fator crucial na tomada de decisões clínicas, havendo necessidade de identificar terapias que promovam controle isquêmico eficaz com menor risco de sangramentos.^3^

O fondaparinux, um inibidor seletivo do fator Xa, demonstrou reduzir a mortalidade e a morbidade em pacientes com SCA em comparação à enoxaparina, principalmente devido à menor incidência de sangramentos.^4,5^ De forma semelhante, o uso do acesso radial para a realização da cinecoronariografia e da ICP tem sido associado à redução de complicações hemorrágicas, conforme demonstrado em estudos observacionais e ensaios clínicos randomizados envolvendo pacientes com SCA.^6,7^ No entanto, os efeitos combinados do fondaparinux e do acesso radial sobre o risco de complicações hemorrágicas e isquêmicas ainda não foram completamente investigados.

O objetivo deste estudo foi avaliar a utilização combinada de fondaparinux e acesso radial em comparação a outras estratégias — fondaparinux com acesso femoral, e enoxaparina com acesso radial ou femoral — em uma coorte de pacientes com SCA na prática clínica. Além disso, buscou-se identificar preditores independentes de melhores desfechos clínicos, com ênfase na terapia antitrombótica e no tipo de acesso arterial utilizado para o cateterismo cardíaco.

## Métodos

### População, Coleta de Dados e Estratégias Terapêuticas

Este é um estudo de coorte retrospectivo, observacional, baseado em registro, realizado em um hospital terciário especializado em doenças cardiovasculares. Desde 2010, todos os pacientes diagnosticados com SCA têm sido continuamente incluídos em um registro como parte do programa institucional de melhoria da qualidade.

Foram incluídos todos os pacientes consecutivos com 18 anos ou mais, diagnosticados com SCA e submetidos a estratégia invasiva entre 1° de janeiro de 2010 e 31 de dezembro de 2017. Considerou-se apenas a primeira internação de cada paciente. Foram excluídos pacientes com infarto agudo do miocárdio (IAM) com supradesnivelamento do segmento ST (IAMCSST) ou IAM tipo II, conforme a Quarta Definição Universal de Infarto do Miocárdio (2018).

Os dados referentes aos desfechos isquêmicos (óbito, IAM e acidente vascular cerebral [AVC]) e eventos hemorrágicos (sangramentos que requereram ou não transfusão ou intervenção cirúrgica) foram coletados prospectivamente durante a hospitalização para fins de controle de qualidade. Informações clínicas adicionais foram extraídas retrospectivamente dos prontuários eletrônicos e posteriormente revisadas por um segundo membro da equipe clínica.

Entre 2010 e 2017, houve uma transição progressiva na prática de anticoagulação na instituição, com a substituição da enoxaparina pelo fondaparinux como anticoagulante preferencial nos pacientes com SCA. Nos pacientes que recebiam fondaparinux, a prática institucional incluía a administração de heparina não fracionada (HNF) na sala de cateterismo durante a ICP.^8^ Para os pacientes tratados com enoxaparina, nenhuma dose adicional de HNF era administrada se a última dose tivesse sido aplicada menos de 8 horas antes do procedimento. No mesmo período, o acesso radial passou a ser a via vascular preferencial para a ICP, com base na publicação de ensaios clínicos randomizados que demonstraram melhores desfechos com essa abordagem.^6,7^

Os pacientes foram agrupados de acordo com o esquema antitrombótico e o tipo de acesso vascular em quatro categorias: fondaparinux + acesso radial, fondaparinux + acesso femoral, enoxaparina + acesso radial e enoxaparina + acesso femoral. A hipótese primária era de que a combinação de fondaparinux com acesso radial estaria associada a melhores desfechos clínicos. O tamanho amostral foi determinado por conveniência.

O desfecho primário foi a ocorrência composta de óbito, reinfarto, AVC ou sangramento maior durante a hospitalização.

O desfecho secundário incluiu o desfecho composto de óbito, reinfarto ou AVC, além das ocorrências isoladas de óbito, reinfarto, AVC, sangramento maior e sangramento total durante a internação.

Sangramento maior foi definido de acordo com uma versão modificada dos critérios do estudo OASIS-5^4^ como qualquer uma das seguintes condições: sangramento fatal, hemorragia intracraniana, hemorragia retroperitoneal, queda da hemoglobina ≥ 2 g/dl com evidência de sangramento, ou qualquer queda de hemoglobina associada a sangramento visível que necessitou de transfusão sanguínea, drogas vasoativas ou intervenção cirúrgica. Episódios de sangramento que não preenchiam os critérios de sangramento maior, mas que levaram à suspensão de agentes antitrombóticos ou se associaram a hematomas > 10 cm, foram classificados como sangramento menor.

### Procedimentos éticos

Este estudo foi conduzido em conformidade com todas as normas nacionais e internacionais aplicáveis à pesquisa envolvendo seres humanos, incluindo a Declaração de Helsinque. O protocolo foi aprovado pelo Comitê de Ética em Pesquisa (CEP) local. Como todos os dados foram anonimizados e extraídos de prontuários médicos e do banco de dados de melhoria da qualidade, o CEP concedeu dispensa do termo de consentimento livre e esclarecido.

### Análise estatística

Os dados foram tabulados e analisados utilizando o software IBM SPSS Statistics for Windows, versão 25.0 (IBM Corp., Armonk, NY, EUA).

As variáveis categóricas foram apresentadas em forma de proporções. As variáveis contínuas foram expressas como média ± desvio padrão (DP) para distribuições paramétricas ou como mediana e intervalo interquartil (IIQ) para distribuições não paramétricas. As variáveis categóricas foram comparadas por meio do teste do qui-quadrado de Pearson. Para variáveis contínuas, utilizou-se o teste *t* de Student não pareado (para dados paramétricos) ou o teste de Mann-Whitney (para dados não paramétricos). Na comparação entre mais de dois grupos, aplicou-se a ANOVA de uma via para variáveis contínuas paramétricas e o teste de Kruskal-Wallis para variáveis contínuas não paramétricas. A normalidade das variáveis contínuas foi avaliada por meio do teste de Kolmogorov-Smirnov e inspeção visual de histogramas.

Após a análise univariada, foi aplicado um modelo de regressão logística multivariada para identificar preditores independentes do desfecho primário. As variáveis incluídas no modelo foram: uso de fondaparinux, acesso radial, o termo de interação entre fondaparinux e acesso radial, além de todas as variáveis com valor de p < 0,10 na análise univariada. O período do estudo não foi incluído no modelo, uma vez que as alterações de protocolo observadas ao longo do tempo estavam intrinsecamente relacionadas ao uso de fondaparinux e ao acesso radial, ambos já contemplados no modelo.

Para comparações envolvendo múltiplos grupos, foi aplicada a correção de Bonferroni. Um valor de p < 0,05 foi considerado estatisticamente significativo para a análise do desfecho primário.

## Resultados

Um total de 956 pacientes foi incluído no estudo. O uso do acesso radial aumentou de 0,7% em 2010 para 67% em 2017. De forma semelhante, o uso de fondaparinux cresceu de 23% para 68% no mesmo período, com pico de 82% em 2014. No total, 661 pacientes receberam fondaparinux e 303 foram tratados com enoxaparina. O acesso radial foi utilizado em 459 pacientes, enquanto o acesso femoral foi empregado em 497.

A Tabela 1 apresenta as características gerais da população total, bem como dos subgrupos definidos pela estratégia anticoagulante e pelo tipo de acesso vascular. A coorte foi composta majoritariamente por homens (54,6%), com média de idade de 65 ± 12,4 anos. Do total, 50,5% foram admitidos com angina instável e 49,5% com IAM sem supradesnivelamento do segmento ST (IAMSSST). As comorbidades incluíram diabetes (33%), hipertensão arterial (75,6%) e dislipidemia (64,7%). Doença arterial coronariana prévia foi relatada em 60% dos pacientes, e 5,6% apresentavam história de AVC.

**Tabela 1:** Características clínicas da população geral e dos subgrupos de acordo com o uso de fondaparinux e a estratégia de acesso vascular

Variável		Total (n = 956)	Fondaparinux + acesso radial (n = 366; 37,9%)	Fondaparinux + acesso femoral (n = 295; 30,6%)	Enoxaparina + acesso radial (n = 93; 9,6%)	Enoxaparina + acesso femoral (n = 202)	Valor p
Sexo masculino, n (%)		526 (55,6%)	206 (56,3%)	153 (51,9%)	52 (55,9%)	110 (54,5%)	0,712
Idade (anos), média ± DP		65,1 ± 12,4	63,2 ± 12,2† *	67,1 ± 11,9§	68,2 ± 12,5§	64,5 ± 13,1	< 0,001
Hipertensão, n (%)		729 (75,6%)	259 (70,8%)†	237 (80,3%)§	77 (82,8%)	150 (74,3%)	0,011
Diabetes, n (%)		321 (33,3%)	111 (30,3%)	111 (37,6%)	32 (34,4%)	61 (30,2%)	0,184
Dislipidemia, n (%)		624 (64,7%)	220 (60,1%)†	209 (70,8%)§	56 (60,2%)	133 (65,8%)	0,027
Insuficiência cardíaca, n (%)		44 (4,6%)	6 (1,6%)†	22 (7,5%)§	6 (6,5%)	10 (5,0%)	0,004
Tabagismo, n (%)		81 (8,4%)	29 (7,9%)	20 (6,8%)	6 (6,5%)	26 (12,9%)	0,080
CRM (pontes de safena), n (%)		154 (16%)	47 (12,8%)†	66 (22,8%)§ *	9 (9,7%)†	31 (15,5%)	< 0,001
ICP prévia, n (%)		128 (13,3%)	37 (10,1%)	44 (15,2%)	18 (19,4%)	27 (13,5%)	0,120
DAC prévia, n (%)		580 (60,7%)	259 (70,8%)† ‡	154 (52,2%)§	54 (58,1%)	113 (55,9%)§	0,001
AVC prévio, n (%)		54 (5,6%)	8 (2,2%)† ‡	25 (8,5%)§	6 (6,5%)	15 (7,4%)§	0,003
	Killip I, n (%)	818 (84,9%)	315 (86,1%)	239 (81,0%)	82 (88,2%)	176 (87,1%)	0,250
**Tipo de SCA**							
	Angina instável, n (%)	487 (50,5%)	192 (52,5%)	148 (50,2%)	49 (52,7%)	96 (47,5%)	0,694
	IAMSSST, n (%)	477 (49,5%)	174 (47,5%)	147 (49,8%)	44 (47,3%)	106 (52,5%)	0,694
	PAS (mmHg), média ± DP	140,4 ± 24,5	142,4 ± 24,8‡	140,3 ± 25,5	139,4 ± 22,5	136,6 ± 22,8§	0,061
	FC (bpm), média ± DP	74,6 ± 18,5	76,1 ± 19,9	73,1 ± 15,9	76,0 ± 19,8	73,3 ± 18,9	0,121
	Fração de ejeção (%), média ± DP	61 ± 12	63 ± 11† ‡	59 ± 13§	61 ± 13	59 ± 13§	< 0,001
	Creatinina (mg/dl), mediana (Q1-Q3)	0,95 (0,61-1,29)	0,90 (0,60-1,20)‡	1,00 (0,60-1,40)	0,91 (0,54-1,28)	1,00 (0,60-1,40)§	< 0,001
	Hemoglobina (g/dl), média ± DP	13,3 ± 1,5	13,6 ± 1,3	13,3 ± 1,6	13,3 ± 1,5	13,2 ± 1,8	0,034
	IMC (kg/m^2^), média ± DP	27,6 ± 4,85	27,7 ± 4,72	27,5 ± 5,03	28,2 ± 5,72	27,4 ± 4,40	0,531
	Tempo de internação (dias), média ± DP	6,7 ± 7,39	5,27 ± 4,47† ‡	7,34 ± 8,72§	6,86 ± 8,61	8,36 ± 8,50§	< 0,001
	Aspirina, n (%)	936 (97,1%)	356 (97%)	287 (97,3%)	87 (93,5%)	198 (98,0%)	0,187
	Tienopiridinas, n (%)	894 (92,7%)	341 (93,2%)*	280 (94,9%)*	73 (78,5%)§ † ‡	192 (95,0%)*	< 0,001
	Inibidor GPIIb/IIIa, n (%)	14 (1,4%)	2 (0,5%)	6 (2,0%)	0 (0%)	6 (3%)	0,110
	ACODs, n (%)	14 (1,4%)	4 (1,1%)*	4 (1,4%)*	6 (6,5%)§ †	4 (2,0%)	0,007
	Varfarina, n (%)	13 (1,3%)	5 (1,4%)	1 (0,3%)*	4 (4,3%)†	3 (1,5%)	0,040
	Acesso radial, n (%)	459 (47,6%)	–	–	–	–	–

§ p < 0,05 vs. Fondaparinux + acesso radial;

† p < 0,05 vs. Fondaparinux + acesso femoral;

* p < 0,05 vs. Enoxaparina + acesso radial;

‡ p < 0,05 vs. Enoxaparina + acesso femoral. As análises de tendência entre os grupos referem-se aos valores p. Variáveis categóricas foram comparadas utilizando o teste do qui-quadrado de Pearson. Variáveis contínuas foram comparadas por ANOVA unidirecional (distribuições paramétricas) ou pelo teste de Kruskal-Wallis (distribuições não paramétricas). ACODs: anticoagulantes orais diretos; AVC: acidente vascular cerebral; CRM: cirurgia de revascularização do miocárdio; DAC: doença arterial coronariana; DP: desvio padrão; GPIIb/IIIa: glicoproteína IIb/IIIa; IAMSSST: infarto do miocárdio sem supradesnivelamento do segmento ST; ICP: intervenção coronariana percutânea; IMC: índice de massa corporal; PAS: pressão arterial sistólica; SCA: síndrome coronariana aguda.

Os pacientes tratados com fondaparinux associado ao acesso radial eram mais jovens e apresentavam menor prevalência de hipertensão, dislipidemia e AVC prévio, além de maior fração de ejeção do ventrículo esquerdo (FEVE), em comparação com aqueles tratados com fondaparinux e acesso femoral. Em relação aos pacientes que receberam enoxaparina com acesso femoral, os tratados com fondaparinux e acesso radial apresentaram menores taxas de insuficiência cardíaca e AVC prévio, menos casos classificados como Killip IV e menor tempo de internação hospitalar. Quando comparados aos pacientes que receberam enoxaparina com acesso radial, os tratados com fondaparinux e acesso radial também eram mais jovens.

**Figura 1:**
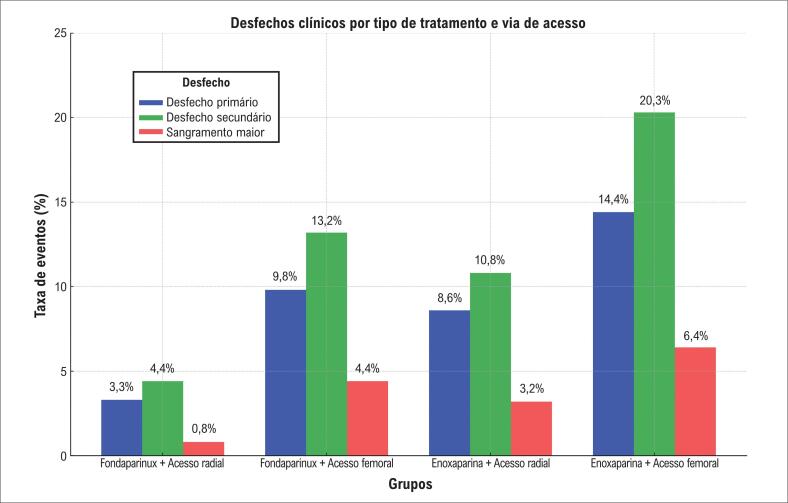
Taxas de eventos clínicos por estratégia de tratamento e via de acesso (fondaparinux ou enoxaparina associados a acesso radial ou femoral). Valor p para tendência < 0,001 para todas as comparações. Desfecho primário: composto por morte hospitalar, reinfarto, AVC ou sangramento maior. Desfecho secundário: composto por morte hospitalar, reinfarto ou AVC. Sangramento maior: definido segundo os critérios modificados do estudo OASIS-5: sangramento fatal; hemorragia intracraniana ou retroperitoneal; queda de hemoglobina ≥ 2 g/dL com evidência de sangramento; ou qualquer queda de hemoglobina com sangramento visível que necessite de transfusão sanguínea, uso de drogas vasoativas ou intervenção cirúrgica. AVC: acidente vascular cerebral.

A Tabela 2 e a Figura 1 mostram as taxas dos desfechos primários e secundários, bem como de seus componentes individuais, tanto para a coorte total quanto para cada grupo de tratamento. O uso de fondaparinux em associação com o acesso radial esteve relacionado às menores taxas de eventos, tanto para o desfecho primário quanto para o secundário. Dentre os componentes individuais, o sangramento maior e a mortalidade foram os que mais contribuíram para essas diferenças observadas.

**Tabela 2:** Desfechos clínicos na população geral e por grupo segundo a estratégia antitrombótica e o acesso vascular

Desfecho	Total (n = 956)	Fondaparinux + acesso radial (n = 366)	Fondaparinux + acesso femoral (n = 295)	Enoxaparina + acesso radial (n = 93)	Enoxaparina + acesso femoral (n = 202)	Valor p
Óbito / reinfarto / AVC / sangramento maior	78 (8,1%)	12 (3,3%) † ‡	29 (9,8%) §	8 (8,6%)	29 (14,4%) §	< 0,001
Óbito / reinfarto / AVC	50 (5,2%)	9 (2,5%) ‡	17 (5,8%)	5 (5,4%)	19 (9,4%) §	0,005
Óbito / reinfarto / AVC / qualquer sangramento	106 (11,0%)	16 (4,4%) † ‡	39 (13,2%) §	10 (10,8%)	41 (20,3%) §	< 0,001
AVC	5 (0,5%)	2 (0,5%)	1 (0,3%)	1 (1,1%)	1 (0,5%)	0,863
Reinfarto	24 (2,5%)	4 (1,1%)	8 (2,7%)	3 (3,2%)	9 (4,5%)	0,095
Sangramento maior	32 (3,3%)	3 (0,8%) † ‡	13 (4,4%) §	3 (3,2%)	13 (6,4%) §	0,003
Sangramento menor	29 (3,0%)	4 (1,1%) ‡	10 (3,4%)	2 (2,2%)	13 (6,4%) §	0,005
Óbito	30 (3,1%)	3 (0,8%) ‡	11 (3,7%)	4 (4,3%)	12 (5,9%) §	0,006

AVC: acidente vascular cerebral. Os valores p referem-se às análises de tendência entre os grupos.

§ p < 0,05 vs. fondaparinux + acesso radial;

† p < 0,05 vs. fondaparinux + acesso femoral;

* p < 0,05 vs. enoxaparina + acesso radial;

‡ p < 0,05 vs. enoxaparina + acesso femoral. As variáveis categóricas foram comparadas pelo teste do qui-quadrado de Pearson.

A Tabela S1 compara os pacientes de acordo com a ocorrência do desfecho primário. Aqueles que apresentaram o desfecho primário tinham maior probabilidade de história prévia de insuficiência cardíaca e AVC, frequência cardíaca mais elevada, menores níveis basais de hemoglobina, FEVE reduzida, níveis elevados de creatinina e maior tempo de internação. O uso de fondaparinux e de acesso radial foi menos frequente entre os pacientes que apresentaram o desfecho primário. Essas variáveis, juntamente com o termo de interação entre fondaparinux e acesso radial, foram incluídas na análise multivariada.

A Tabela 3 apresenta os preditores independentes do desfecho primário. O uso de fondaparinux esteve associado à redução de 43% na ocorrência do desfecho primário (OR 0,57; IC 95% 0,34-0,96; p < 0,05). O acesso radial também foi associado independentemente à redução de 54% nesse desfecho (OR 0,46; IC 95% 0,26-0,83; p = 0,01). Os níveis de creatinina, a FEVE e o tempo de internação também foram identificados como preditores independentes. Não foi observada interação significativa entre o uso de fondaparinux e o acesso radial (p_interação_ = 0,56).

**Tabela 3:** Preditores independentes do desfecho primário (análise de regressão logística multivariada)

Variável	OR (IC 95%)	Valor de p
Revascularização prévia	0,98 (0,56–1,72)	0,95
Hemoglobina basal	0,98 (0,84–1,13)	0,79
Diabetes	0,86 (0,50–1,50)	0,61
Insuficiência cardíaca	1,67 (0,68–4,11)	0,26
AVC prévio	1,58 (0,70–3,58)	0,26
Creatinina	1,15 (0,99–1,33)	0,06
Fondaparinux mais acesso radial	0,69 (0,21–2,26)	0,56
Fondaparinux (isoladamente)	0,57 (0,34–0,96)	0,03
Acesso radial (isoladamente)	0,46 (0,26–0,83)	0,01
Fração de ejeção	0,04 (0,008–0,264)	< 0,001
Duração da internação (dias)	1,06 (1,038–1,098)	< 0,001

Desfecho primário: composto de morte hospitalar, reinfarto, AVC ou sangramento maior. AVC: acidente vascular cerebral.

## Discusssão

Nesta coorte de mundo real composta por pacientes com SCA, tanto o uso de fondaparinux quanto o acesso radial estiveram associados, de forma independente, a uma menor incidência do desfecho composto — óbito, reinfarto, AVC e sangramento maior — durante a hospitalização. Observou-se um possível efeito aditivo, uma vez que os pacientes tratados com fondaparinux e acesso radial apresentaram as menores taxas de eventos em comparação com outras combinações de estratégias antitrombóticas e vias de acesso vascular (Figura Central).

Em pacientes com IAMSSST, estudos prévios demonstraram que o fondaparinux não é inferior à enoxaparina em relação aos MACE, sendo superior na redução de sangramentos e mortalidade em 30 dias, conforme evidenciado no estudo OASIS-5.^4^ Além disso, seu uso em associação à HNF durante o procedimento de cateterismo não parece aumentar o risco de sangramento.^8^ Esses achados foram confirmados por análises de registros clínicos tanto na Suécia quanto no Brasil.^9,10^ Nossos resultados corroboram esses dados: a taxa de sangramento maior foi de apenas 1% entre os pacientes tratados com fondaparinux + acesso radial, e de 4,4% entre aqueles com fondaparinux + acesso femoral. No registro sueco, a taxa global de sangramento com fondaparinux foi de 1,1%, embora não tenha havido estratificação por tipo de acesso vascular.

O acesso radial tem sido associado à redução de sangramentos e eventos adversos em ensaios clínicos multicêntricos randomizados^7^ e em metanálises.^11^ Embora um estudo prévio não tenha demonstrado esses benefícios, diferenças na experiência dos operadores e nas taxas de eventos podem justificar essa discrepância. As diretrizes clínicas atuais recomendam o acesso radial como via preferencial.^12^ No entanto, nenhum desses estudos estratificou os desfechos conforme a terapia antitrombótica utilizada.

Em nossa coorte, o acesso radial foi associado, de forma independente, à redução de eventos isquêmicos e hemorrágicos, com efeito mais pronunciado nos pacientes tratados com fondaparinux. O possível benefício aditivo da combinação entre acesso radial e determinados agentes antitrombóticos permanece pouco explorado. Mina et al.^13^ em metanálise que avaliou bivalirudina + acesso radial, observaram que o benefício em termos de sangramento ocorreu apenas com o uso de acesso femoral, sem benefício adicional com o uso do acesso radial em relação ao MACE. Em contraste, nossos achados sugerem que o fondaparinux confere benefício independentemente da via de acesso, mas que a adição do acesso radial resulta em risco ainda menor de desfechos adversos.

Almendro-Delia et al., ao analisarem uma coorte na Andaluzia (Espanha), identificaram uma interação positiva entre fondaparinux e acesso radial. Na análise exploratória do estudo, o fondaparinux mostrou benefício apenas no subgrupo com acesso radial, sem vantagem observada no subgrupo com acesso femoral.^14^ Em nosso modelo multivariado, tanto o fondaparinux quanto o acesso radial mantiveram associação independente com menor risco do desfecho primário. Isso reforça a hipótese de efeito independente e aditivo, independentemente da via de acesso — semelhante ao observado nos estudos pivôs com fondaparinux, nos quais o acesso utilizado foi predominantemente femoral.^4,5^

Nosso estudo apresenta algumas limitações. Primeiramente, trata-se de uma análise retrospectiva e unicêntrica. No entanto, os eventos clínicos foram monitorados prospectivamente como parte de um protocolo institucional de melhoria da qualidade. Em segundo lugar, optamos por utilizar os critérios de sangramento do estudo OASIS-5 em vez da classificação mais recente do BARC,^15^ a fim de manter a comparabilidade com a literatura específica sobre fondaparinux. Em terceiro lugar, o acompanhamento foi limitado ao período de internação. No OASIS-5,^4^ benefícios adicionais foram observados aos 30 dias, o que pode explicar as taxas relativamente baixas de eventos em nossa coorte e influenciar a interpretação dos resultados relacionados ao acesso radial. Por fim, apesar dos ajustes realizados, persistem diferenças basais entre os grupos. Embora uma análise com pareamento por escore de propensão pudesse minimizar esse viés, optamos por não a realizar devido à significativa redução amostral que acarretaria.

Estudos futuros devem contemplar acompanhamento em longo prazo para investigar se a combinação entre fondaparinux e acesso radial oferece benefícios sustentados ao longo do tempo.

## Conclusão

A utilização de fondaparinux em associação ao acesso radial esteve associada a menores taxas de MACE e de sangramento maior em pacientes com SCA. Ensaios clínicos randomizados futuros ou estudos com pareamento por escore de propensão bem delineados poderão contribuir para elucidar melhor esse possível benefício aditivo.

## Data Availability

Dados podem ser disponibilizados com análise caso a caso, de acordo com a justificativa e após aprovação ao comitê científico do estudo.

## *Material suplementar

Para informação adicional, por favor, clique aqui.
