# Supplementary material for: Associação entre o Uso de Fondaparinux com Acesso Radial e Desfechos Clínicos em Pacientes com Síndrome Coronariana Aguda sem Supradesnivelamento do Segmento ST
Source: Arq Bras Cardiol. 2025 Nov 13;122(9):e20240329. [Article in Portuguese] doi: 10.36660/abc.20240329 (PMC12671839; doi:10.36660/abc.20240329)
Supplement: Translation

# Translation: Association between the Use of Fondaparinux Plus Radial Access and Clinical Outcomes in Patients with Non-ST Elevation Acute Coronary Syndrome

**DOI:** 10.36660/abc.20240329i

**Authors:** Luiz Eduardo Fonteles Ritt, Eduardo Sahade Darze, Pedro Gabriel Melo de Barros e Silva, Gilson Soares Feitosa-Filho, João Victor Santos Pereira Ramos, Márcia A. Viana, Priscila Neri Lacerda, Emanoela Lima Freitas, Queila Oliveira Borges, Adriano Oliveira Martins, Renato Delascio Lopes

**Affiliations:** 1 Salvador BA Brazil D’Or Research Institute, Hospital Cárdio Pulmonar, Salvador, BA – Brazil; 2 Salvador BA Brazil Escola Bahiana de Medicina e Saúde Pública, Salvador, BA – Brazil; 3 São Paulo SP Brazil Hospital Samaritano Paulista, São Paulo, SP – Brazil; 4 Salvador BA Brazil Universidade Federal da Bahia, Salvador, BA – Brazil; 5 Durham North Carolina USA Duke University Hospital, Durham, North Carolina – USA

**Keywords:** Acute Coronary Syndromes, Cardiac Catheterization, Prognosis

## Abstract

**Background:**

Both fondaparinux and radial access have been associated with lower rates of major adverse cardiovascular events (MACE) in acute coronary syndrome (ACS).

**Objective:**

To evaluate the association between the use of fondaparinux plus radial access and clinical outcomes.

**Methods:**

In this study, 956 patients admitted with ACS and treated with an invasive strategy were analyzed. The primary outcome — a composite of major bleeding (according to OASIS-5 criteria) and MACE — was compared across groups defined by anticoagulation regimen (fondaparinux or enoxaparin) plus arterial access site (femoral vs. radial). A p-value < 0.05 was considered statistically significant.

**Results:**

The mean age of the study population was 65 ± 12.4 years, and 49.5% presented with non-ST segment elevation myocardial infarction (NSTEMI). Fondaparinux and radial access were used concurrently in 366 patients. The primary endpoint occurred in 78 patients (8.1%): MACE in 50 (5.2%) and major bleeding in 32 (3.3%). The event rate was lowest in the fondaparinux plus radial access group (3.3%), compared with enoxaparin plus radial access (9.8%), fondaparinux plus femoral access (8.6%), and enoxaparin plus femoral access (14.4%) (p < 0.001). Multivariable analysis showed that the use of fondaparinux was associated with a 43% reduction in the primary outcome (OR, 0.57; 95% CI, 0.34-0.96; p < 0.05), and radial access was independently associated with a 54% reduction (OR, 0.46; 95% CI, 0.26-0.83; p = 0.01).

**Conclusion:**

The combination of fondaparinux and radial access was associated with the lowest rates of MACE and major bleeding, compared to either strategy alone.

## Introduction

Acute coronary syndromes (ACS) are among the leading causes of death worldwide. Over the past two decades, the treatment of ACS has evolved to incorporate an aggressive combination of anticoagulant and antiplatelet agents, along with an early invasive strategy involving coronary angiography and percutaneous coronary intervention (PCI), when indicated. As a result, the risk of ischemic complications has significantly decreased; however, this has been accompanied by a noticeable increase in bleeding risk.^1–3^

Bleeding events in patients with ACS are associated with worse clinical outcomes, including higher mortality rates. Consequently, bleeding risk has become a key consideration in clinical decision-making, and there is a need to identify therapies that provide effective ischemic control while minimizing bleeding complications.^3^

Fondaparinux, a selective factor Xa inhibitor, has been shown to reduce mortality and morbidity in patients with ACS when compared to enoxaparin, primarily due to a lower incidence of bleeding.^4,5^ Similarly, the use of radial access for coronary angiography and PCI has been associated with a reduction in bleeding complications, as demonstrated in both observational studies and randomized trials involving patients with ACS.^6,7^ Nevertheless, the combined effects of fondaparinux and radial access on the risk of hemorrhagic and ischemic complications have not been thoroughly investigated.

**Figure:** ACS: acute coronary syndrome; MACE: major adverse cardiovascular events.

The aim of this study was to evaluate the combined use of fondaparinux and radial access compared with other strategies — fondaparinux with femoral access and enoxaparin with either radial or femoral access — in a real-world cohort of patients with ACS. Additionally, we sought to identify independent predictors of better clinical outcomes, with a particular focus on antithrombotic therapy and the type of arterial access used for cardiac catheterization.

## Methods

### Population, Data Collection, and Treatment Strategies

This retrospective, observational, registry-based cohort study was conducted at a tertiary care hospital specializing in cardiovascular disease. Since 2010, all patients diagnosed with ACS have been continuously enrolled in a registry as part of the institution's quality improvement program.

We included all consecutive patients aged 18 years or older who were diagnosed with ACS and underwent an invasive strategy between January 1, 2010, and December 31, 2017. Only the first hospitalization was considered for each patient. Patients with ST-segment elevation myocardial infarction (STEMI) or type II acute myocardial infarction (AMI), as defined by the 2018 Fourth Universal Definition of Myocardial Infarction, were excluded.

Data on ischemic outcomes (death, AMI, and stroke) and hemorrhagic events (bleeding episodes requiring or not requiring transfusion or surgical intervention) were prospectively collected during hospitalization for quality control purposes. Additional clinical data were retrospectively retrieved from electronic medical records and subsequently reviewed by a second member of the clinical team.

Between 2010 and 2017, there was a progressive transition in anticoagulation practice at the institution, shifting from enoxaparin to fondaparinux as the preferred anticoagulant for patients with ACS. In patients receiving fondaparinux, the routine practice included administering unfractionated heparin (UFH) in the catheterization laboratory during PCI.^8^ For patients treated with enoxaparin, if the last dose had been administered less than 8 hours before the procedure, no additional UFH was given. Over the same period, radial access became the preferred vascular approach for PCI, following the publication of randomized trials demonstrating superior outcomes with this technique.^6,7^

Patients were grouped according to antithrombotic treatment and vascular access site into four categories: fondaparinux with radial access, fondaparinux with femoral access, enoxaparin with radial access, and enoxaparin with femoral access. The primary hypothesis was that the combination of fondaparinux and radial access would be associated with better clinical outcomes. Sample size was determined by convenience.

The primary endpoint was the composite occurrence of death, reinfarction, stroke, or major bleeding during hospitalization.

The secondary endpoint included the composite of death, reinfarction, or stroke as well as the individual occurrences of death, reinfarction, stroke, major bleeding, and total bleeding during the hospitalization period.

Major bleeding was defined according to a modified version of the OASIS-5 trial criteria^4^ as any of the following: fatal bleeding, intracranial hemorrhage, retroperitoneal hemorrhage, a hemoglobin drop of ≥ 2 g/dL with evidence of bleeding, or any hemoglobin drop with visible bleeding requiring blood transfusion, vasoactive drugs, or surgical intervention. Bleeding episodes that did not meet the major bleeding criteria but were associated with either withdrawal of antithrombotic agents or hematomas > 10 cm were classified as minor bleeding.

### Ethical procedures

This study was conducted in accordance with all applicable national and international regulations governing research involving human participants, including the Declaration of Helsinki. The study protocol was approved by the local Institutional Review Board (IRB). As all data were anonymized and obtained from medical records and a quality improvement database, the IRB granted us a waiver of informed consent.

### Statistical analysis

Data were tabulated and analyzed using IBM SPSS Statistics for Windows, version 25.0 (IBM Corp., Armonk, N.Y., USA).

Categorical variables were reported as proportions. Continuous variables were expressed as mean ± standard deviation (SD) for parametric distributions or as median and interquartile range (IQR) for nonparametric distributions. Categorical variables were compared using Pearson's chi-square test. Continuous variables were compared using the unpaired Student's *t*-test (for parametric data) or the Mann-Whitney *U* test (for nonparametric data). When comparing more than two groups, one-way ANOVA was used for parametric continuous variables and the Kruskal-Wallis test for nonparametric variables. The normality of continuous variables was assessed using the Kolmogorov-Smirnov test and visual inspection of histograms.

Following univariable analysis, a multivariable logistic regression model was applied to identify independent predictors of the primary endpoint. Variables included in the model were the use of fondaparinux, radial access, the interaction term between fondaparinux and radial access, and any variable with a p-value < 0.10 in univariable analysis. The study period was not included in the model, as the observed protocol changes over time were inherently linked to the use of fondaparinux and radial access, both of which were already accounted for in the model.

For comparisons involving multiple groups, the Bonferroni correction was applied. A p-value < 0.05 was considered statistically significant for the primary endpoint analysis.

## Results

A total of 956 patients were included in the study. The use of radial artery access increased from 0.7% in 2010 to 67% in 2017. Similarly, fondaparinux use rose from 23% to 68% during the same period, peaking at 82% in 2014. Overall, 661 patients received fondaparinux and 303 received an enoxaparin-based strategy. Radial access was used in 459 patients, while femoral access was used in 497.

Table 1 presents the general characteristics of the overall population, as well as subgroups based on anticoagulant strategy and access site. The cohort had a slight male predominance (54.6%), and the mean age was 65 ± 12.4 years. Of the total population, 50.5% were admitted with unstable angina and 49.5% with NSTEMI. Comorbidities included diabetes (33%), hypertension (75.6%), and dyslipidemia (64.7%). Prior coronary artery disease was reported in 60% of patients, and 5.6% had a history of stroke.

**Table 1:** Clinical characteristics of the overall population and subgroups according to fondaparinux use and vascular access strategy

Variable		Total (n = 956)	Fondaparinux plus radial access (n = 366; 37.9%)	Fondaparinux plus femoral access (n = 295, 30.6%)	Enoxaparin plus radial access (n = 93, 9.6%)	Enoxaparin plus femoral access (n = 202)	p-value
Male sex, n (%)		526 (55.6%)	206 (56.3%)	153 (51.9%)	52 (55.9%)	110 (54.5%)	0.712
Age (years), mean ± SD		65.1 ± 12.4	63.2 ± 12.2† *	67.1 ± 11.9§	68.2 ± 12.5§	64.5 ± 13.1	< 0.001
Hypertension, n (%)		729 (75.6%)	259 (70.8%)†	237 (80.3%)§	77 (82.8%)	150 (74.3%)	0.011
Diabetes, n (%)		321 (33.3%)	111 (30.3%)	111 (37.6%)	32 (34.4%)	61 (30.2%)	0.184
Dyslipidemia, n (%)		624 (64.7%)	220 (60.1%)†	209 (70.8%)§	56 (60.2%)	133 (65.8%)	0.027
Heart failure, n (%)		44 (4.6%)	6 (1.6%)†	22 (7.5%)§	6 (6.5%)	10 (5.0%)	0.004
Smoking, n (%)		81 (8.4%)	29 (7.9%)	20 (6.8%)	6 (6.5%)	26 (12.9%)	0.080
CABG, n (%)		154 (16%)	47 (12.8%)†	66 (22.8%)§ *	9 (9.7%)†	31 (15.5%)	< 0.001
Prior PCI, n (%)		128 (13.3%)	37 (10.1%)	44 (15.2%)	18 (19.4%)	27 (13.5%)	0.120
Prior CAD,		580 (60.7%)	259 (70.8%)† ‡	154 (52.2%)§	54 (58.1%)	113 (55.9%)§	0.001
Prior stroke, n (%)		54 (5.6%)	8 (2.2%)† ‡	25 (8.5%)§	6 (6.5%)	15 (7.4%)§	0.003
	Killip I, n (%)	818 (84.9%)	315 (86.1%)	239 (81.0%)	82 (88.2%)	176 (87.1%)	0.250
**Type of ACS**							
	Unstable angina, n (%)	487 (50.5%)	192 (52.5%)	148 (50.2%)	49 (52.7%)	96 (47.5%)	0.694
	NSTEMI, n (%)	477 (49.5%)	174 (47.5%)	147 (49.8%)	44 (47.3%)	106 (52.5%)	0.694
	SBP, (mmHg) mean ± SD	140.4 ± 24.5	142.4 ± 24.8‡	140.3 ± 25.5	139.4 ± 22.5	136.6 ± 22.8§	0.061
	Heart rate (bpm), mean ± SD	74.6 ± 18.5	76.1 ± 19.9	73.1 ± 15.9	76.0 ± 19.8	73.3 ± 18.9	0.121
	Ejection fraction (%), mean ± SD	61 ± 12	63 ± 11† ‡	59 ± 13§	61 ± 13	59 ± 13§	< 0.001
	Creatinine (mg/dL), median (Q1-Q3)	0.95 (0.61-1.29)	0.90 (0.60-1.20)‡	1.00 (0.60-1.40)	0.91 (0.54-1.28)	1.00 (0.60-1.40)§	< 0.001
	Hemoglobin (g/dL), mean ± SD	13.3 ± 1.5	13.6 ± 1.3	13.3 ± 1.6	13.3 ± 1.5	13.2 ± 1.8	0.034
	BMI (kg/m^2^), mean ± SD	27.6 ± 4.85	27.7 ± 4.72	27.5 ± 5.03	28.2 ± 5.72	27.4 ± 4.40	0.531
	Length of hospital stay (days), mean ± SD	6.7 ± 7.39	5.27 ± 4.47† ‡	7.34 ± 8.72§	6.86 ± 8.61	8.36 ± 8.50§	< 0.001
	Aspirin, n (%)	936 (97.1%)	356 (97%)	287 (97.3%)	87 (93.5%)	198 (98.0%)	0.187
	Thienopyridines, n (%)	894 (92.7%)	341 (93.2%)*	280 (94.9%)*	73 (78.5%)§ † ‡	192 (95.0%)*	< 0.001
	GPIIb/IIIa inhibitor, n (%)	14 (1.4%)	2 (0.5%)	6 (2.0%)	0 (0%)	6 (3%)	0.110
	DOACs, n (%)	14 (1.4%)	4 (1.1%)*	4 (1.4%)*	6 (6.5%)§ †	4 (2.0%)	0.007
	Warfarin, n (%)	13 (1.3%)	5 (1.4%)	1 (0.3%)*	4 (4.3%)†	3 (1.5%)	0.040
	Radial access, n (%)	459 (47.6%)	–	–	–	–	–

§ p < 0.05 vs. Fondaparinux plus radial access;

† p < 0.05 vs. Fondaparinux plus femoral access;

* p < 0.05 vs. Enoxaparin plus radial access;

‡ p < 0.05 vs. Enoxaparin plus femoral access. P-values refer to trend analyses between groups. Categorical variables were compared using Pearson's chi-square test. Continuous variables were compared using one-way ANOVA for parametric distributions or the Kruskal-Wallis test for nonparametric distributions. ACS: acute coronary syndrome; BMI: body mass index; CABG: coronary artery bypass grafting; CAD: coronary artery disease; DOACs: direct oral anticoagulants; GPIIb/IIIa: glycoprotein IIb/IIIa; NSTEMI: non-ST-elevation myocardial infarction; PCI: percutaneous coronary intervention; SD: standard deviation; SBP: systolic blood pressure.

Patients treated with fondaparinux plus radial access were younger and had a lower prevalence of hypertension, dyslipidemia, and prior stroke as well as higher left ventricular ejection fraction (LVEF) compared with those treated with fondaparinux plus femoral access. Compared to patients who received enoxaparin and femoral access, those in the fondaparinux plus radial group had lower rates of heart failure and previous stroke, fewer cases in Killip class IV, and shorter hospital stays. When compared with patients who received enoxaparin plus radial access, those treated with fondaparinux plus radial access were younger.

Table 2 and Figure 1 show the rates of primary and secondary endpoints, along with their individual components, for the overall cohort and each treatment group. The use of fondaparinux plus radial access was associated with the lowest event rates for both the primary and secondary endpoints. Among the individual components, major bleeding and death contributed most to the observed differences.

**Table 2:** Clinical outcomes in the overall population and by group according to antithrombotic strategy and vascular access

Outcome	Total (n = 956)	Fondaparinux plus radial (n = 366)	Fondaparinux plus femoral (n = 295)	Enoxaparin plus radial (n = 93)	Enoxaparin plus femoral (n = 202)	p-value
Death / reinfarction / stroke / major bleeding	78 (8.1%)	12 (3.3%) † ‡	29 (9.8%) §	8 (8.6%)	29 (14.4%) §	< 0.001
Death / reinfarction / stroke	50 (5.2%)	9 (2.5%) ‡	17 (5.8%)	5 (5.4%)	19 (9.4%) §	0.005
Death / reinfarction / stroke / any bleeding	106 (11.0%)	16 (4.4%) † ‡	39 (13.2%) §	10 (10.8%)	41 (20.3%) §	< 0.001
Stroke	5 (0.5%)	2 (0.5%)	1 (0.3%)	1 (1.1%)	1 (0.5%)	0.863
Reinfarction	24 (2.5%)	4 (1.1%)	8 (2.7%)	3 (3.2%)	9 (4.5%)	0.095
Major bleeding	32 (3.3%)	3 (0.8%) † ‡	13 (4.4%) §	3 (3.2%)	13 (6.4%) §	0.003
Minor bleeding	29 (3.0%)	4 (1.1%) ‡	10 (3.4%)	2 (2.2%)	13 (6.4%) §	0.005
Death	30 (3.1%)	3 (0.8%) ‡	11 (3.7%)	4 (4.3%)	12 (5.9%) §	0.006

*P*-values refer to trend analyses across groups.

§ p < 0.05 vs. fondaparinux plus radial access;

† p < 0.05 vs. fondaparinux plus femoral access;

* p < 0.05 vs. enoxaparin plus radial access;

‡ p < 0.05 vs. enoxaparin plus femoral access. Categorical variables were compared using Pearson's chi-square test.

**Figure 1:** Clinical event rates by treatment and access strategy (fondaparinux or enoxaparin plus radial or femoral access). P-value for trend < 0.001 for all comparisons. Primary outcome: composite of in-hospital death, reinfarction, stroke, or major bleeding. Secondary outcome: composite of in-hospital death, reinfarction, or stroke. Major bleeding: defined according to modified OASIS-5 trial criteria: fatal bleeding; intracranial or retroperitoneal hemorrhage; hemoglobin drop of ≥ 2 g/dL with evidence of bleeding; or any hemoglobin drop with visible bleeding requiring blood transfusion, vasoactive agents, or surgical intervention.

Table S1 compares patients according to the occurrence of the primary endpoint. Those who experienced the primary outcome were more likely to have a history of heart failure and stroke, a higher heart rate, lower baseline hemoglobin levels, decreased LVEF, elevated creatinine levels, and longer hospital stays. Fondaparinux and radial access were less frequently used among patients who experienced the primary endpoint. These variables, along with the interaction term between fondaparinux and radial access, were included in the multivariable analysis.

Table 3 shows the independent predictors of the primary endpoint. The use of fondaparinux was associated with a 43% reduction in the occurrence of the primary outcome (OR, 0.57; 95% CI, 0.34-0.96; p < 0.05). Radial access was also independently associated with a 54% reduction (OR, 0.46; 95% CI, 0.26-0.83; p = 0.01). Creatinine level, LVEF, and length of hospital stay were also identified as independent predictors. No significant interaction was found between fondaparinux use and radial access (p_interaction_ = 0.56).

**Table 3:** Independent predictors of the primary endpoint (multivariable logistic regression analysis)

Variable	OR (95% CI)	p-value
Previous revascularization	0.98 (0.56–1.72)	0.95
Baseline hemoglobin	0.98 (0.84–1.13)	0.79
Diabetes	0.86 (0.50–1.50)	0.61
Heart failure	1.67 (0.68–4.11)	0.26
Prior stroke	1.58 (0.70–3.58)	0.26
Creatinine	1.15 (0.99–1.33)	0.06
Fondaparinux plus radial access	0.69 (0.21–2.26)	0.56
Fondaparinux (alone)	0.57 (0.34–0.96)	0.03
Radial access (alone)	0.46 (0.26–0.83)	0.01
Ejection fraction	0.04 (0.008–0.264)	< 0.001
Length of hospital stay	1.06 (1.038–1.098)	< 0.001

Primary endpoint: composite of in-hospital death, reinfarction, stroke, or major bleeding.

## Discussion

In this real-world cohort of patients with ACS, both fondaparinux and radial access were independently associated with a lower incidence of the composite endpoint — death, reinfarction, stroke, and major bleeding — during hospitalization. An additive effect was suggested, as patients treated with fondaparinux plus radial access experienced the lowest event rates when compared with other combinations of antithrombotic strategy and vascular access (Central Illustration).

In patients with NSTEMI, fondaparinux has previously been shown to be noninferior to enoxaparin in terms of MACE and superior in reducing bleeding events and 30-day mortality, as demonstrated in the OASIS-5 trial.^4^ Moreover, its recommended use alongside UFH in the catheterization laboratory does not appear to increase bleeding risk.^8^ These findings have been confirmed in registry analyses from both Sweden and Brazil.^9,10^ Our results are consistent with these findings: the major bleeding rate in our study was as low as 1% in patients treated with fondaparinux plus radial access, and 4.4% in those treated with fondaparinux plus femoral access. In the Swedish registry, the overall bleeding rate with fondaparinux was 1.1%; however, no stratification by access site was reported.

Radial access has been associated with reduced bleeding and lower event rates in randomized multicenter trials^7^ and meta-analyses.^11^ While an earlier trial^6^ failed to confirm these benefits, differences in operator experience and event rates may explain the discrepancy. Current clinical guidelines recommend radial access as the preferred approach.^12^ However, none of these trials stratified outcomes by antithrombotic therapy.

In our cohort, radial access was independently associated with fewer bleeding and ischemic events, and this effect appeared more pronounced in patients treated with fondaparinux. The potential additive benefit of radial access combined with specific antithrombotic agents remains underexplored. Mina et al.^13^ evaluated bivalirudin plus radial access in a meta-analysis and found that the bleeding benefit of bivalirudin was significant only with femoral access; no additional benefit was observed with radial access in terms of MACE. In contrast, our findings suggest that fondaparinux confers benefit regardless of access route, but that the addition of radial access yields an even lower risk of adverse outcomes.

Almendro-Delia et al. analyzed a cohort in Andalusia, Spain, and found a positive interaction between fondaparinux plus radial access. In their exploratory analysis, fondaparinux remained beneficial only in the radial access subgroup, with no observed advantage in the femoral subgroup.^14^ In our multivariable model, both fondaparinux and radial access remained independently associated with reduced risk of the primary outcome. This supports the hypothesis of an independent and additive effect, regardless of access route — similar to what was observed in the pivotal fondaparinux trials, where femoral access was predominantly used.^4,5^

Our study has several limitations. First, it was a retrospective, single-center analysis. However, clinical events were tracked prospectively as part of a quality improvement protocol. Second, we chose to use the bleeding criteria from the OASIS-5 trial rather than the more recently proposed BARC definitions,^15^ to maintain consistency with literature on fondaparinux. Third, our follow-up was limited to the in-hospital period. In OASIS-5,^4^ additional benefit was observed at 30 days, which may explain the relatively low event rates in our cohort and influence the interpretation of radial access outcomes. Finally, despite adjustments, baseline differences between groups were present. Although a propensity score matching analysis could have minimized this bias, we opted against it due to the substantial reduction in sample size it would entail.

Future studies should focus on a longer follow-up period to determine whether the combination is more beneficial over the long term.

## Conclusion

The use of fondaparinux plus radial access was associated with lower rates of MACE and major bleeding in patients with ACS. Future randomized trials or well-designed propensity-matched studies may help to better elucidate this potentially additive benefit.

## Data Availability

Data may be made available on a case-by-case basis, depending on the justification and after approval by the study's scientific committee.

## *Supplemental Materials

For additional information, please click here.
